# Women’s Empowerment, Agency and Self-Determination in Afrobeats Music Videos: A Multimodal Critical Discourse Analysis

**DOI:** 10.3389/fsoc.2021.646899

**Published:** 2021-05-03

**Authors:** Simphiwe Emmanuel Rens

**Affiliations:** Department of Communication Science, University of South Africa, Pretoria, South Africa

**Keywords:** agency, empowerment, self-determination, post-feminism, misogyrom, afrobeats, midriff sensibility

## Abstract

Stemming from a broader PhD project, this article argues that neoliberal post-feminist cultural sensibilities–entrenched in contemporary popular culture–about empowered, agentic and self-determining women, are regressive for the feminist advancement of gender-relational equality in the African context. To arrive at this conclusion, the central aim was to elucidate whether the gender-performative representations prioritised in the multimodal discourses of Afrobeats music videos are implicated in post-feminist sensibilities and if so, in what ways and to what effect? Given the continent’s richly diverse, yet largely heteropatriarchal, sociocultural formations, I argue that ideas about empowered, agentic and self-determining (black) African women are–based on the limited purview offered through the multimodal discourses of a small corpus of Afrobeats music videos–no more than sociocultural façades as opposed to gender-relational realities in our context. The article relied on a multimodal critical discourse analysis of a total of nine music videos drawn from the PhD project’s larger corpus of 25 Afrobeats music videos, their accompanying song lyrics, as well as a selection of YouTube viewer comments extracted from the analysed music videos. In critically exploring the gender-relational depictions prioritised in the analysed music videos, I argue for the consideration of what I am coining “misogyrom”; a gender-relational cultural sensibility which, in tandem with a post-feminist sensibility partly undergirding the multimodal discourses of these music videos, effectively veil this popular musical genre’s evidently sexist and misogynistic undertones that subvert potentialities of empowered, agentic and self-determining black African women.

## Introduction

Afrobeats–a globally popularising African musical genre–is widely consumed by transnational audiences. This sees the music videos produced by artists from this musical genre, reach YouTube viewership figures ranging in the hundreds of millions. As such, these Global South popular cultural texts are valuable conduits through which to critically examine contemporary social constructions and performances of gender and politically charged meanings of “womanhood” in relation to “manhood” as implicated as they are in heteropatriarchal collective African cultural worldviews. This article situates this musical genre’s multimodal discourses, specifically as they relate to gender and sexuality, within a broader post-feminist discourse about women’s (sexual) empowerment, agency and self-determination. The central aim, thus, is to elucidate whether the gender-performative representations prioritised in the multimodal discourses of Afrobeats music videos are implicated in post-feminist sensibilities and if so, in what ways and to what effect? I follow Simidele Dosekun in my considerations of post-feminist sensibilities in that I, like Dosekun (2015), regard post-feminism as a transnational cultural sensibility. Thereby, also regarding it an analytically relevant and valuable scholarly lens in the context from which this current article emerges: an African popular cultural landscape. Relying on a multifaceted conceptual framework, the article argues that a close reading of this musical genre’s multimodal discourses through a post-feminist lens exposes the ill-convincing nature–in African popular cultural texts–of neoliberal, post-feminist cultural sensibilities at pains with positioning (heterosexual) women as sexually empowered, agentic and self-determining as concerns their relations with men across variously heteropatriarchal African sociocultural formations.

Incorporated in this study’s conceptual framework are feminist scholarly contributions about gender as performance (Butler, 1988; Butler, 1990), the (heteropatriarchal) male gazes and their implications on constructions of feminine subjectivities (Mulvey, 1975; Mulvey, 1989; Gill, 2007; Gill, 2008), as well as literature pertaining to the “midriff” post-feminist sensibility in popular media forms (Gill, 2008). These scholarly contributions aid this current article in articulating its arguments with respect to gender and sexuality as variously performed across the selection of Afrobeats music videos–stemming from a larger corpus from my PhD project–analysed in this study. By so doing, the article is able to demonstrate how a notable post-feminist sensibility–encouraging notions of women’s empowerment, agency and self-determination as it relates to sex and sexuality–specifically undergirds the performances of femininities within these music videos. However, these readings of women’s sexual empowerment, agency and self-determination, I will argue, are mere social façades that are effectively mediated and pseudo-legitimised through discourses of extreme (heteronormative) romanticism from diverse black African masculinities which–if taken at face value–may encourage the uncritical absolving of hetero-patriarchy from its deeply toxic nature toward black African women’s actual sociocultural advancement and just treatment.

This article draws specifically on nine music videos selected from a larger corpus of 25 Afrobeats music videos (from my PhD project) with high viewership figures–as at November 2018, when said music videos were downloaded from YouTube. These nine music videos form the core corpus of data analysed in this research article. In addition, for each of the nine music videos sampled, a written transcript of the song’s lyrics was also subjected to critical analysis. As secondary data, a textual corpus of YouTube viewer comments from each of the nine analysed music videos was also critically analysed. The article deployed a multimodal critical discourse analysis of the select Afrobeats music videos, their accompanying song lyrics, as well as the YouTube viewer comments so as to develop its arguments.

Structurally, this article consists of four main sections following this introductory section. The first part situates the study’s empirical object of analysis within Judith-Butler-inspired scholarship about gender performativity, as well as scholarly literature on post-feminism. Thereafter, I offer a critical reading of performances of “manhood”/masculinities in the analysed texts in order to demonstrate dominant constructions of “manhood” in the hyperreal gender-relational contexts mediated in the analysed music videos. By so doing, I am able to briefly demonstrate three dominant forms of performative masculinities pervasive in the analysed multimodal discourses. It is in this section where I propose a new gender-relational cultural sensibility I call “misogyrom”—a cultural sensibility that I argue mediates performances of femininities that intersect with a salient post-feminist sensibility as evident in the analysed music videos.

In the third section, I engage a critical reading of performances of “womanhood”/femininities in the analysed music videos. My reliance on a multifaceted conceptual framework undergirded by post-feminist cultural sensibilities, aids me in highlighting possible readings of women’s empowerment, agency and self-determination in the multimodal discourses of this musical genre. I achieve this by drawing on plot analyses from some randomly selected music videos from the nine analysed in this article. Lastly, in the section following this one, I advance an argument that readings of post-feminist notions of women’s empowerment, agency and self-determination in Afrobeats music videos–as African popular cultural texts–are mere social façades. Façades that are mediated and pseudo-legitimised by discourses of extreme (heteronormative) romanticism, implicated in a misogyrom sensibility which–if taken at face value–may inspire the uncritical absolving of heteropatriarchy from its deeply toxic nature toward black African women’s *actual* sociocultural advancement and just treatment, more especially in the domains of sexual expression.

## Gender, Gender Performativity, and a Post-Feminist Sensibility in Afrobeats Music Videos

Gender performativity, as proposed by Judith Butler, purports that gender is a ritualised performance across diverse human societies. Butler (1988) argues broadly that gender identities are socially constructed notions that affect our self-perceptions about who we believe we are as subjectivities of a particular biological sex. For Butler (1988); Butler (1990), the distinction between biological sex and gender is crucial in demonstrating her argument that genders–unlike sex–are socioculturally constructed and performed as opposed to being physiobiological facts determined by the possession of particular reproductive genitalia (vagina or penis). Butler’s constructionist view of gender identity underscores her argument in this regard. Essentially, this view argues that in accordance with political, historical and social values and ideas, gender is actively and, to an extent, unwittingly, constructed by human subjectivities who learn and cling to particular behavioral cues that are relied upon to craft what we come to widely accept as women’s and men’s behaviors (Butler, 1988).

These learnt behaviors, in turn, are inextricably linked to notions of femininity and masculinity as themselves co-constructed along psychosocial influences of what it means to be born in possession of a particular external genital organ. With regard to gender, so argues Butler (1988: 524) argues:

Because there is neither an”essence” that gender ex-presses or externalizes nor an objective ideal to which gender aspires; because gender is not a fact, the various acts of gender creates the idea of gender, and without those acts, there would be no gender at all. Gender is, thus, a construction that regularly conceals its genesis.

It is by acknowledging the distinction between gender and sex that we can observe the constructionist implications of gender in its performative guize. Gender identities are, thus, in constant flux and states of “becoming” as soon as one is born into this world. Commenting on how “real” or factual our gender identities are, (Butler 1988: 524) reminds us that “[g]ender reality is performative which means, quite simply, that it is real only to the extent that it is performed. The influences of diverse societies’ social, political, cultural and historical ideological regimes on the kinds of “acts” associated with “manhood” and “womanhood” in said societies, perpetuate certain kinds of acts that are widely interpreted as being expressive of a gender identity. These acts are normalised as inherently linked to biological sex, and conformity to said acts is implicitly celebrated as “natural”.

What this does, is it enforces “sedimented expectations of gendered existence” (Butler, 1988: 524). Significantly inspired by parts of Simone de Beauvoir’s theoretical contributions, Butler’s influential body of work on gender performativity problematizes these sedimented expectations of gendered existence by exposing how the body becomes

…its gender through a series of acts which are renewed, revised, and consolidated through time. From a feminist point of view, one might try to reconceive the gendered body as the legacy of sedimented acts rather than a predetermined or foreclosed structure, essence or fact, whether natural, cultural, or linguistic.

Genders, as socially constructed notions, Butler argues, can then be perceived as neither true nor false, neither real nor apparent; but ever-evolving performative regimes that variously compel us to “act” in certain ways depending on our possession of either a penis or vagina.

In this current article, I borrow from this body of work on gender as a performative social construction. The article closely links these theoretical insights to my critical approach to the corpus of Afrobeats music videos collected for this study. These music videos, as sites of gender representation, expose us to creatively crafted, active constructions and performances of gender (and sexuality). That is to say, the processes of gender identity construction are salient in these music videos due to how these texts deploy multimodal devices to establish, maintain, contest and/or problematize gender “norms”. Creators of these music videos are actively involved in narrating and, thereby, idealizing particular gender performances to foreground various points of views about what it means to be a woman or a man across the richly diverse African and diasporic contexts captured in this musical genre’s visual culture.

**FIGURE 1:**
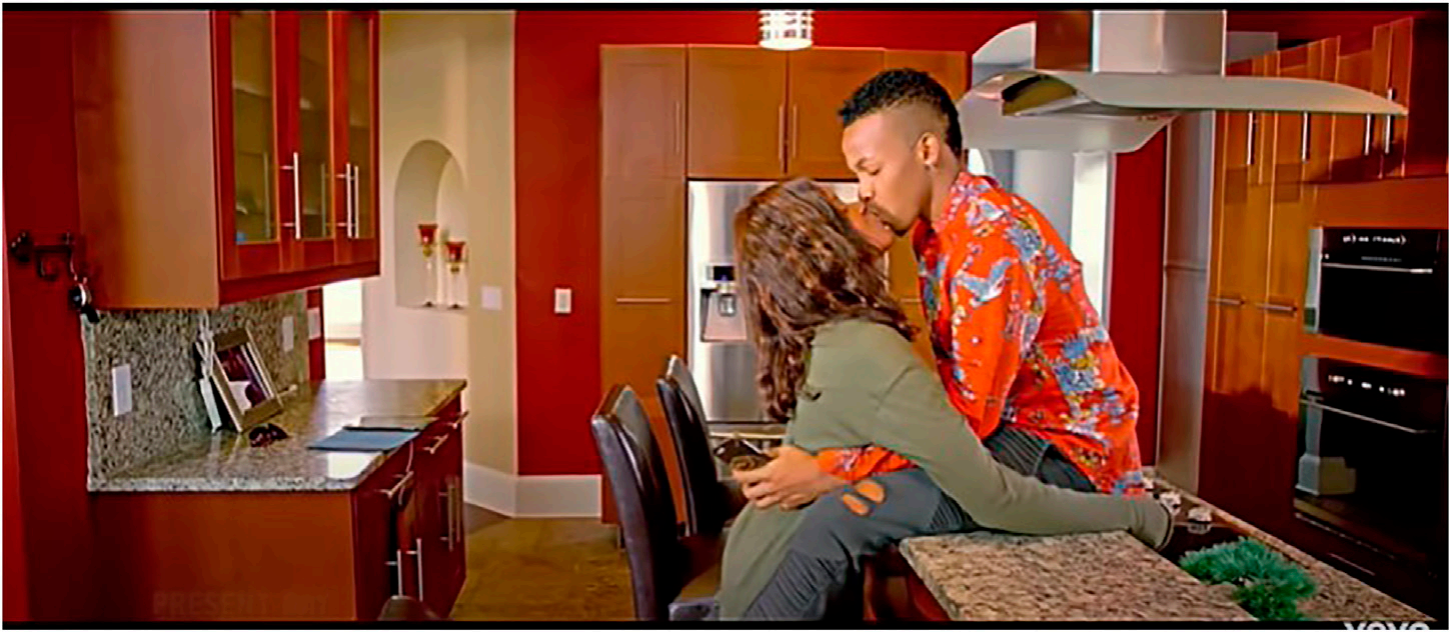
Visual display of heterosexual romantic relations in Tekno’s “Where” music video [screenshot taken from *Where (Official Music Video):* https://www.youtube.com/watch?v=ooFAF18L5wQ].

**FIGURE 2:**
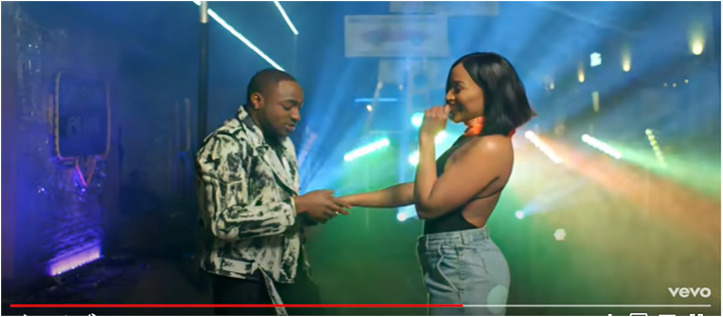
Visual display of heterosexual romantic relations in Davido’s “If” music video [screenshot taken from *If (Official Music Video):* https://www.youtube.com/watch?v=helEv0kGHd4].

This study’s analysis of the data in relation to gender performativity, reveals that a heteronormative worldview underscores the multimodal representations prevalent in these texts. On the part of the featured African women and men, gender performativity and gender relations in the music videos analyzed, are saliently heterosexual-oriented, considering the visual and lyrical contents of these music videos. Consider the visual depictions in Figures 1, 2 where an explicit heterosexual romantic orientation is sustained through the denotative depictions of romantic love and intimacy via non-verbal gestures such as kissing, romantic caressing and other forms of intimate physical touch. Evident here, is a striking visual trope wherein the predominant expressions of sexuality are overtly aligned along normative ideals that overtly celebrate heterosexuality. As such, African “womanhood” and “manhood”, for instance, are exclusively imagined through this limited purview.

Let us closely consider Miles (2016b) music video for the song, “Where” (from which Figure 1 is extracted). The entire plot revolves around a heterosexual couple in love, and this love is emphasised through several scenes such as the one depicted here. This brightly lit scene captures Tekno and his love interest sharing a kiss while in romantic embrace with one another. The kiss is shared in what appears to be a domestic setting of a kitchen (given typical kitchen furniture in clear view), suggesting that the couple happily live together. The brightness of this scene implies that the act depicted therein is something to be illuminated, foregrounded, and not hidden in the dark. In that vein, it is suggested that romantic heterosexual expressions of sexuality are to be celebrated, lauded, and aspired to; sustaining, thus, the normative hegemonic dominance that heteronormativity enjoys in contemporary (African) societies. In extension–when considered through a post-feminist lens–such depictions also affirm to women watching these music videos that heterosexual expressions of femininity and female sexuality are to be aspired to if such romantic “happiness” is to be achieved. That is, in order to be romantically pursued and mesmerised by attractive, sexually alluring men (as evidenced in Figure 2, for instance), a woman should knowingly (thus, agentically?) present a “shy”, yet aesthetically enticing, version of herself in public spaces in order to “capture” the man of her interest. I will argue, then, that it is critical to expose the catalytic nature of masculine performative expressions in popular culture on performances and constructions of femininities. As such, in the next section, I will be offering a critical analysis of performances of masculinities in Afrobeats music videos, as they are implicated in encouraging particular “ways of seeing” (Berger, 1990) femininities in said music videos. But first, a conceptualization of post-feminism as undergirding this paper’s critical analyses. Feminist plights–in their diverse iterations over time and context–toward the just treatment of women, have variously been implicated in rather contradictory principles that seem to situate feminism almost as something of the past; a plight/sensibility no longer required (McRobbie 2004). This discourse has had an influential role in the pervasiveness of a sensibility called “post-feminism” (McRobbie 2004; Gill 2007; Gill 2008). What postfeminist-aligned “worldviews” do, in the context of popular culture, is that they variously undermine the feminist gains of the 1970's and 80’s, argues Angela McRobbie (2004). McRobbie, as Storey (2018: 176) summarizes, makes no secret of her low optimism about the presumed ‘success’ of feminism:

What has really happened, she argues, is that much contemporary popular culture actively undermines the feminist gains of the 1970’s and 1980’s. However, this should not be understood as a straightforward ‘backlash’ against feminism. Rather its undermining of feminism works by acknowledging feminism while at the same time suggesting that it is no longer necessary in a world where women have the freedom to shape their own individual life courses. In post-feminist popular culture feminism features as history: aged, uncool and redundant. The acknowledging of feminism, therefore, is only to demonstrate that it is no longer relevant.

It is through this advancement of a perception that feminism is no longer relevant, that in contemporary popular culture, we are bombarded with countless discourses (visual or otherwise) that actively position women as empowered, agentic, and self-determining among other things. A postfeminist sensibility is deeply entrenched in a neoliberal worldview that calls for the celebration of individuality and choice said to be unrestrictedly afforded to women globally (Gill, 2007, Gill, 2008; Dosekun, 2015). This sensibility “…is less contingent upon political or organized activism than the feminist of the past” (Cuklanz, 2016: 9), and in relation to expressions of sexuality, more specifically, women are believed–and increasingly represented in contemporary popular media forms–as enjoying sexual freedom and free expression of sexuality (Gill, 2007; Gill, 2008; Genz, 2009; Cuklanz, 2016).

From such discursive formations about women’s sexual freedom and free expression of sexuality as indicative of a postfeminist consciousness, emerged critical womanist scholarship that have theorised women as “midriff” (Gill, 2007; 2008) to conceptualise women’s witting use of their bodies, appearances, and sexualities to more insistently exercise their sexual agency. Midriff women are described as unapologetic about their sexual power and ability to (freely) use their bodies as means to socioculturally, and otherwise, advance or benefit themselves (Gill, 2007; Gill, 2008). It will be interesting, then, to trace existing inscriptions of the post-feminist sensibility in a Global South produced media text: the Afrobeats music video, as this is an area currently less explored.

## Tracing “Misogyrom”: A Critical Reading of Performances of Masculinities in Afrobeats Music Videos

Anyone doing a surface-based random online search for music videos from the Afrobeats musical genre will witness that it is notably male-dominated. The leading artists and performers in this scene are predominantly black African men. As such, the music video storylines and song lyrics produced from this musical genre engender an unapologetic masculine aura that significantly shapes the multimodal discourses of this music, as they relate to gender relations. In this section, I outline three dominant forms of masculine performativities by the male artists in the corpus of music videos analysed in this article. I unpack performances of “breadwinner” masculinities (Groes-Green, 2009), misogynistically “sexualised” masculinities, as well as romance-oriented masculinities and their intersections with performances of femininities in the analysed music videos.

The protagonist men centralised in the analysed music videos, are depicted as unquestionably wealthy and ideal ‘breadwinners’ who assert their dominance over women by “parading” their wealth through crass consumerist depictions of themselves. In very pervasive fashions, the men featured in the music videos analysed for this paper, perform manhood through various materialistic, provision-driven foundations of spending largely as a means to emphasise powerful manhood. Let us consider the song “If” by Davido (2017b), for instance. A consumerist, breadwinner-underpinned, performance of manhood is sustained through the lyrical content wherein he pledges to his love interest in the song that he will take care of her financially by giving her money and adorning her body with expensive, luxurious clothing brands such as Versace and Gucci. Davido says:

If I tell you say I love you o

My money my body na your own o baby

Thirty billion for the account o

Versace and Gucci for your body o baby

Here, Davido expresses that, in declaring his love for this woman, she must know that his money and body are hers. He assures her of his wealth status by emphasising that his bank account will have as much as 30 billion, from which this woman should expect to be spoiled with luxury fashion brands such as Versace and Gucci. Here, an implicit suggestion is advanced which positions the woman, in the mind of the viewer, as being incapable of securing her own financial means to access the luxurious commodities she is lured with by the man. An incapability arguably brought on by many reasons, including but not limited to, a sexist heteronorm-conforming belief pertaining to gender roles: that women (in their limited capacities as “wives” and “homemakers”) are not expected to partake in the active accrual of wealth as this role is reserved for the male figure who shall act as main provider/breadwinner. Herein, I argue, we can begin to see misogyny rearing its regressive head.

Thus, at play here, is the partial upkeep of misogynistically mediated gender relations that aid men in their preservation of cultural power over women through economic superiority (Mills and Ssewakiryanga 2005). Such gender relations–mediated by men-owned money and material commodities–perpetuate the patriarchal ideologies and structures responsible for the subordination of women across various sociocultural domains, including the domains of (heterosexual) sexuality/sexual expression, intimate and romantic relationships.

Further to that, men’s dominance is also partly sustained through hyper-sexualized, partly misogynistic expressions of manhood in the analyzed music videos. These sexualized masculinities are socially constructed in ways that encourage men’s authority and power (in relation to women) by anchoring powerful manhood on notions of sexual performance and perceived sexual prowess. Afrobeats musician, Tekno, engages in the promotion of a particularly sexualized masculine performartivity. The lyrical contents from his song “Pana” (Miles, 2016a) are largely about sex and love (The Biafran, 2016). We shall, nonetheless, just consider an excerpt from the song in the interest of space.

[…]

Baby, Pana

Anywhere that you go

I go follow you dey go

Baby, Pana

They say you like cassava

I getti big cassava

Baby, Pana

My love for you will never die, will never die

Herein, Tekno deploys an infantilizing tone–pervasive in much of the lyrical content analyzed in this study–to endearingly address the woman he refers to as ‘Pana’ in the song. He goes on to assure the woman that anywhere she goes, he will follow her before he admits that it has come to his attention that this woman enjoys consuming cassava (a cassava is an elongated, cone-shaped, brown root vegetable usually of notable length and girth). It is with this knowledge about her apparent like for cassava, that Tekno informs her that he is in possession of a big cassava, and then finally proclaims his undying love for this woman.

This musician engages in the promotion of a sexualized masculine expression that is sustained through lyrics portraying him as a man who is in state of loving and sexually satisfying his love interest. Here, Tekno deploys a food item as a metaphor to refer to the penis, particularly. Due to her said fondness for penis, Tekno makes it known to this woman that he has a large penis; seemingly attempting to convince her that he makes for an ideal sexual partner, as a result of this claim. Herewith, Tekno demonstrates a representational trope within the Afrobeats music videos analyzed, that emphasizes and maintains particularly sexualized masculinities expressed by men from socioculturally diverse African backgrounds in the service of male supremacy. Such pursuits, I suggest, uphold men’s superiority and a misogynistic undermining of women as merely passive receivers of men’s (sexual) provision and pleasure when considered through the domain of sexual expression and intimate relationships.

This kind of creative language use to indicate sexual prowess on the part of the men in the corpus of music videos analyzed in this paper, is not lost on consumers of this music. As evidenced in the corpus of YouTube comments analyzed alongside the music videos, viewers reproduce these discourses in the comments section; legitimizing and elevating the sexualized masculinities perpetuated in these music videos.

“Looks like Tekno has given Cassava a whole new meaning. Lol. Loving this song so far.” (username: “Maka Velli”)

“828 niggas who can't provide big cassava for their bebs [“babes”] dislike this life saving song 😂 Y'all should pana, Tekno gat big cassava for y'all bebs [“babes”].” (username: “RElindid Ayuk”)

“Only guys with big Cassava like this comment !” (username: “PassyPom Official”)

“am totally looking for someone with a big cassava not a carrot please help me out” (username: “Carol C”)

Here, we can see how men are either superiorized or inferiorized based on perceptions of penis size–which is directly linked to sexual satisfaction. “Maka Velli”—who humorously (given the use of the acronym “LOL,” meaning “laughing out loud”) shares–that s/he loves this song, makes us aware that the artist has repurposed the meaning of the word cassava. As suggested above, Tekno"s use of the word in this song, has been sexualized; thus, dissociating it from its intended food-related, literal meaning.

Accepted, by viewers of this music video, as a symbol of a man’s sexual organ, the cassava is used by “RElindid Ayuk” to ridicule men who are perceived to be unable to live up to the size expectations linked to the penis in relation to women (or “babes”, as this viewer refers to them). According to this viewer, when they were writing this comment on YouTube, there were “828 niggas [in some black youth cultures, the term “nigga” is sometimes casually used to refer to men]” who disliked the music video because they are unable to present a large enough penis to their lovers/women, risking the chance of losing said women to the likes of men such as Tekno who claim to have large penises. It is through this kind of reasoning that hierarchized forms of masculinities are sustained in the minds of consumers of these music videos; perpetuating, as a result, divisive comments which celebrate a particular kind of superiority of some men over others. In this case, it is suggested that the most desirable man is “…someone with a big cassava [large penis] not a carrot [smaller penis]…“, as “Carol C” expressly shares.

Further evidence of a sexualized, partly misogynistic, masculine expression on the part of the featured men over the featured women’s bodies, can be observed in the lyrics excerpt below. The following lyrical contents are taken form Davido’s second verse in the song, “Aye” (Davido, 2014):

The baddest!

Baby girl you the baddest

Oya shakee your asset

Make e man no go forget

Referring to her as “baby girl” in what is, yet again, evidence of an infantilising tone, Davido appeals to the woman to “shakee your asset”; using colloquial English to instruct the lady that she must gyrate her “assets”. The word “asset”, in this context, refers to the woman’s posterior or buttocks. For doing what is asked of her, the woman is hailed as “the baddest”. Her “badness” is emphasised in a positive manner, expressing warranted badness that is encouraged by Davido who claims that by being “the baddest”, the woman will be unforgettable to men, specifically. She is unforgettably good in her expression of ‘badness’ to a point where, by shaking her buttocks as requested by a man, she will be able to “[m]ake e man no go forget”—this pidgin English phrase can be translated as an affirmation to the woman that her movement of her “assets” will render her memorable or unforgettable to a man—in this case, Davido. This woman’s expression of her femininity is at once hypersexualised and controlled by a male other who, by getting her to oblige to his misogynistic instructions, exercises dominance over her. Relegating her to a position of servitude in her femininity. This is a position of servitude that unlocks several perks and benefits for the complying woman. Most significant of all, is social capital attached to mere proximity to the masculinities in these music videos, constructed as particularly wealthy, sexually potent, and emotionally transparent about their undying love for the women they pursue.

Very strikingly, however–and in notable dissent to existing literature on masculinities in popular (black) male-dominated musical genres such as rap and hip-hop–the multi-faceted expressions of masculinities evident in the music videos analyzed for this article, engender a very overt romanticism intricately implicated in notable counter-stereotypical work, encouraging somewhat of a myth-debunking potential. This romanticism is mediated by a particularly gentle and non-violent overtone that renders these masculinities physically non-threatening to the femininities with which the screen is shared. Therefore, in spite of their implicit misogynistic behaviors, the observed masculinities appear to equally value genuine romantic love built on foundations of fidelity.

Acts of romantic love and commitment–in their very explicitly heteronorm-conforming iterations–are consistently depicted in the Afrobeats music videos analyzed in this paper. To exemplify this, let us consider the narrative progression of the song “Bank Alert” by P-Square (2016). The music video predominantly makes use of three modes of representation to communicate a romantic love story between a boyfriend (played by a member of P-Square) and his supposed girlfriend. These modes are visual, textual and oral.

Visually–in the opening scene of the music video–the couple are depicted as underprivileged Nigerian youngsters having a conversation against the backdrop of what appears to be a property that still requires renovation as the plastering on the walls appears to be patchy and unfinished (communicating financial/economic struggle). The filmic technique of a black-and-white visual filter adds to emphasize this sense of underprivilege or poverty. In this briefly mediated oral conversation, the man is informing the woman that he needs to go abroad for a year so as to go and better his life. He then makes a promise to the woman that he will be back in one year’s time especially for her, and then reassures her of his love for her. The almost inaudible background music layered beneath the visuals in this scene, includes lyrics such as “I belong to you, you belong to me … And I’ll be missing you, missing you yeah … “. The amalgamation of these various modes gives the scene a slight air of innocent love and romance.

To communicate a time lapse in the narrative, written text is used over the image-based visuals of what appears to be moving clouds to inform the viewer that about five years have passed since that conversation between the two young lovers had unfolded. This time, a colour-based visual filter is used by the editors of this music video to signify recency and, to an extent, a change in the narrative. What happens next in the narrative–supported by the lyrical content of the song–is indicative of a portrayal of a particularly romance-oriented masculine expression on the part of the protagonist man in the music video. Although not within the time range promised, the man eventually *does* return to Nigeria and is adamant to ask for his girlfriend’s hand in marriage to demonstrate his undying love for her. He is now visibly affluent (denoted by scenes of him traveling back in a private jet). And thus, pledges his love for her while promising to financially take care of this woman (true to form to the kind of breadwinner-based masculinities prevalent in the music videos analyzed) should she say yes to his marriage proposal. Affluence, it seems, is deployed here as a tangible catalytic mode to express romanticism. Below, I closely consider the following excerpt of lyrics in Pidgin English from this song:

Today na today eh,

and when I finish you go no say na me

As you dey they delay eh, to proof my love, you must to chop my money eeh

Across several of the richly diverse cultures in Nigeria, when a couple are set to get married, a date is set for when the fiancé–with his family and friends–are expected to conspicuously expend money and gifts to his parents-in-law-to-be while the bride-to-be watches on at a distance. Through the lyrics presented above, Paul (who portrays the protagonist) boasts about his excitement for that day where he will “proof” his love.

This, to an extent, communicates that the African men in these music videos possess an endearing regard for women as more than just objects of their sexual desires. The multimodal discourses evident in the Afrobeats music videos analyzed, socially construct and sustain such romance-oriented masculinities that thrive on manhood being expressed through grand romantic gestures toward women. The culturally and linguistically diverse African men depicted in these music videos are largely portrayed (especially through the song lyrics) as commitment-driven, family oriented, loyal and capable of offering infinite romantic love to the women they get romantically involved with, as long as these women successfully conform to conventional gender roles which allow men’s dominance over them. Seemingly at play here, then, are striking forms of benevolently sexist and implicitly misogynistic advances of men’s superiority that are partly veiled by, and mediated through, romanticism.

It is against this backdrop, that I argue for the consideration of a gender-relational cultural sensibility I am coining misogyrom. This term combines the concepts of “misogyny” and “romanticism” to argue for the reading of gender relations that present a notable masking of misogynistic expressions of “manhood” with overdramaticized forms of romanticism toward women. I thus also propose the term as a conceptual lens through which to consider men’s (and by extension, women’s) performativities that are at once implicated in the perpetuation of misogynistic imbalanced gender power relations and undeniable hyper-romanticism and compassion from (heterosexual) men toward women. Should we apply the concept of misogyrom in the context of the music video texts analyzed in this paper, it is apparent that idealized (heterosexual) expressions of “manhood” are sustained along inherently sexist and misogynistic expressions of men’s superiority and dominance while (in)advertently encouraging the egalitarian treatment of women by valuing them as more than mere objects of men’s sexual satisfaction, but romantic partners with value more than that which a patriarchal male gaze had historically often relegated to *just* physical appearance. This, I argue, presents the social construction of masculinities implicated in a misogyrom sensibility.

The misogyrom-aligning masculinities pervasive in the analyzed music videos, present a striking dialectic which, when closely analyzed, is much more regressive than beneficial to the feminist plight toward gender equality, in the domains of, but not limited to, sexuality and intimacy. The two sides to this dialectic involve extreme romanticism on the one hand, and notable misogyny on the other. In their active visual and lyrics-based overdramatisations of romanticism through their endearing lyrics and storylines, symbolizing genuine emotional connectedness to women beyond superficial goals to score sexual favors, the masculinities in these music videos garner adulation from consumers of these texts. These comments of adulation, from YouTube viewers of these music videos, laud these men as the epitomes of exemplary (African) manhood while being oblivious to the ways in which these masculine expressions maintain patriarchy and misogyny.

“You guys did our culture justice in this video! This should be a lesson to all the young men who make a success of their lives! Come back home and get your chocolate bride!!!!!!! LOVE YOU ALL FOR THIS VIDEO!!!!!!” (username: Kwini Souleimane)

“While African singers are singing love and much more for the black woman, our bother and sis in the US OF A are denigrating black woman....” (username: PHONE master)

In their comment on the music video for the song “Bank Alert” by P-Square, “Kwini Souleimane,” expresses what appears to be an appreciation toward P-Square for “doing justice” to their culture (presumably, Igbo culture–given the fact that the men making up the musical ensemble of P-Square are ethnically of Igbo descent). This viewer goes on to suggest that the contents of this music video capture a lesson that “all young men who make a success of their lives” should learn. The lesson being, that “all young men” who embark on greener pasture pursuits away from home, should return–once financially secure–and “get” their bride. While evidently heteropatriarchal and sexist in its suggestion that women are objects that men should “get” and lead into the heteronormative institution of marriage, this viewer’s comment makes us aware that they find such kinds of gestures from men to be romantic. Such romantic gestures by the Igbo, or Yoruba, or African men in the music videos analyzed, are lauded because they show that “[w]hile African singers are singing love and much more for the black woman, our bother and sis in the US OF A are denigrating black woman”, shares another viewer. Thus, placing black African men, in the minds of viewers of these Afrobeats music videos, in a favourable position as being non-violent toward women, romantic and full of love.

It is precisely the overdramatisations of romanticism in these music videos that conceal the dark side of the dialectic sensibility of misogyrom. As a result, what partly prevails is an effective distraction from the misogyny these masculinities equally embody which, when left unchallenged, may perpetuate the naive absolving of patriarchy from its toxic nature in relation to the just and empowering sociocultural treatment of women across various sociocultural domains, but most especially the domains of sexuality and intimate relations. Even so, are possibilities of women’s empowerment non-existent within the multimodal discourses of the Afrobeats musical genre? Below, I discover–through a post-feminist lens–that the picture is perhaps not so bleak.

## Empowered, Agentic and Self-Determining African Women? A Post-feminist Reading of Performative Femininities in Afrobeats Music Videos

In their capacities as “featured characters” across the music video texts analyzed for this paper, women’s bodies depicted alongside the male musicians dominating this musical genre, are variously subjected to black African male gazes/”looks”. Acts of looking have been theorized as powerful and value-laden. As such, the notion of “the gaze” is weaved into the conceptual framework of this article given the visual nature of the study’s primary data. Gazing/looking is, in psychoanalytic terms, a key aspect involved in the construction of particular subjectivities and their sexual and gender identity formations. This gaze, to follow Mulvey (1975); Mulvey (1989), partly takes on power-related manifestations through fetishism and voyeurism. Fetishism, from a psychoanalytic perspective, refers to a process whereby a viewing subject displaces their (sexual) energy, “urges,” and desires onto another object outside said viewing subject (Hall, 2013: 256). This is done as a means to (sub)consciously confront laden fantasies that the viewing subject may be “faced with” on a psychological level. Due to the intangible nature of desire-driven fantasy, an individual physicalizes their desires, curiosities, and urges, by using another physical object to represent those desires, curiosities and urges (Hall, 2013: 256).

The “object” that eventually becomes the replacement of the looker’s unseen, intangible desires and fantasies, is gazed upon as a reassuring, non-threatening, passive object of display; strategically positioned as a spectacle “in an intimate relation to the spectator” (Rose, 2001: 111). In the context of film, as an example, creators deploy certain filmic techniques to encourage a fetishistic gaze. These include, among others, the use of camera angles: framing through close-up shots, medium-shots, wide-shots and deliberately timed shots that often exclude everything from the viewer’s gaze except the body, or parts of the body (buttocks, bosom, thigs, face etc) of, more often, the female characters (Rose, 2001). The Afrobeats music videos analyzed in this paper–as themselves short, film-like narrative-driven texts–similarly deploy filmic techniques to spatially position women in ways that encourage notable fetishistic gazes over said women’s bodies often by the domineering black African men pervasive in these texts. Thereby, fostering a sense of voyeurism on the part of said men featured in these music videos, as well as the viewers actively consuming these texts across various screens.

Voyeurism, as simplified by Rose (2001; drawing from Mulvey, 1989), refers to a way of actively looking at a distanced object that is objectified by the looker, at a distance ensuring that the viewed object is not aware of this gaze. This gaze possesses a certain control that may, at times, even be sadistic in its motif (Rose, 2001). In film, this is a look often exclusively given to (heterosexual) men, whether as characters in the film or as audience member viewing the film (Rose, 2001: 110). Mulvey’s early postulations (1975; 1989) argued that voyeurism is produced by the visio-spatial organization of a film, relying on particular tools of compositional interpretation (Rose, 2001: 110). Such tools of compositional interpretation include, and are not limited to, the placing of (physical) distance between the male and female protagonists of a film (or music video, in the context of this article); as well as placing (physical) distance between the female protagonist of a film/music video and the audience (Rose, 2001: 110).

Considering the three “kinds of look (ing)” that visual texts promote: 1) the look stemming from the camera to the event/scene; 2) the look existing between the characters portrayed through the action in, for the purpose of this study, the music videos; 3) as well as the look stemming from the spectator to the ‘action’ on screen (Nixon et al., 2013: 314), broader sociocultural implications that said (gendered) “kinds of looking” have on the social construction of particular expressions of ‘femaleness’ as a gender performance, may be interrogated (Butler, 1990).

Across the corpus of analyzed music videos, the camera persistently assumes the position of the gazer/spectator who is seemingly imagined to be a heterosexual man, able to derive visual and erotic pleasure from fetishizing parts of the women’s bodies hypervisibilized in these music videos. By strategically focusing on, and directing viewer attention to focus predominantly on specific body parts of the women (instead of the women’s bodies as a whole), Afrobeats music video directors and editors sustain this fetishizing culture because “[the] substitution of a *part* for the *whole,* of a thing–an object, an organ, a portion of the body–for a *subject*, is the effect of a very important representational practice–*fetishism*” (Hall, 2013: 256).

Women’s buttocks, their lower body parts: hips, thighs/legs and inguinal/groin area and breast area are subjected to close-up shots that overtly drive viewer attention to these body parts that are socio-culturally understood and accepted as sexual body parts. By so doing, spectators of this visual simulacra are afforded the opportunity to take part in this fetishistic culture that (sexually) objectifies the woman’s body for the satisfaction of (heterosexual) male gazes. This way, viewers may arguably be invited to glean erotic satisfaction, among other things, through their engagement with these Afrobeats music videos.

Perhaps unsurprisingly, the conversational tones undergirding the analyzed viewer comments do not problematize this noted sexual objectification of black women’s bodies in the analyzed music videos. Instead, viewers expressly celebrate the hypersexualized women’s bodies in these texts. Consider comments contributed by viewers such as “d_won1” who comments on black women’s physical appearance by posing the question, “Why are Black women just so gorgeous? #melaninpoppin.” Other such comments include those by viewers such as “Lei Shay,” “Daniel Shamu,” and “Barwaago Aden” who, similarly, pass commentary that directly speak to the perceived beauty of black women:

“omg, black girls are so beautiful” (username: “Lei Shay”)

“Black women are beautiful fam damn” (username: “Daniel Shamu”)

“too much beauty in one video 😍❤😩. black sisters are slaying 🔥.” (username: “Barwaaqo Aden”).”

These comments explicitly link these viewers’ perceptions of beauty to the black women’s bodies hypersexualized in these music videos. The sexually objectified black women in the music videos are described in ways that not only emphasize their physical appearance, but at times also places them on a symbolic pedestal that linguistically portrays them as the epitome of beauty to a point where they are described as magical and perfect “creations.” See, for instance, the extracted comments from viewers below:

“The girls in this video are all GOALS! Black women, we're a gift to the world!” (username: “Simply Shanice”)

“Black women are one of God's most perfect creations🔥 #blackgirlmagic” (username: “being sashyka”)

Viewers are expressive about their admiration for the featured black women and their perceived beauty. We can see, then, how a culture of sexualized fetishism by creators of these music videos is successfully internalized by viewers of these music videos.

This process of fetishistic objectification as an expression of internal (sexual) desires emerges dominantly in the Afrobeats music videos analyzed for this research paper. These fetishistic gazes bestowed particularly upon women’s bodies are sustained through the encouragement of a voyeuristic way of looking at the women featured in these music videos. The Afrobeats music videos analyzed in this paper highlight particularly active black African male gazes that variously objectify women’s bodies by actively distancing themselves from the bodies being look at.

**FIGURE 3:**
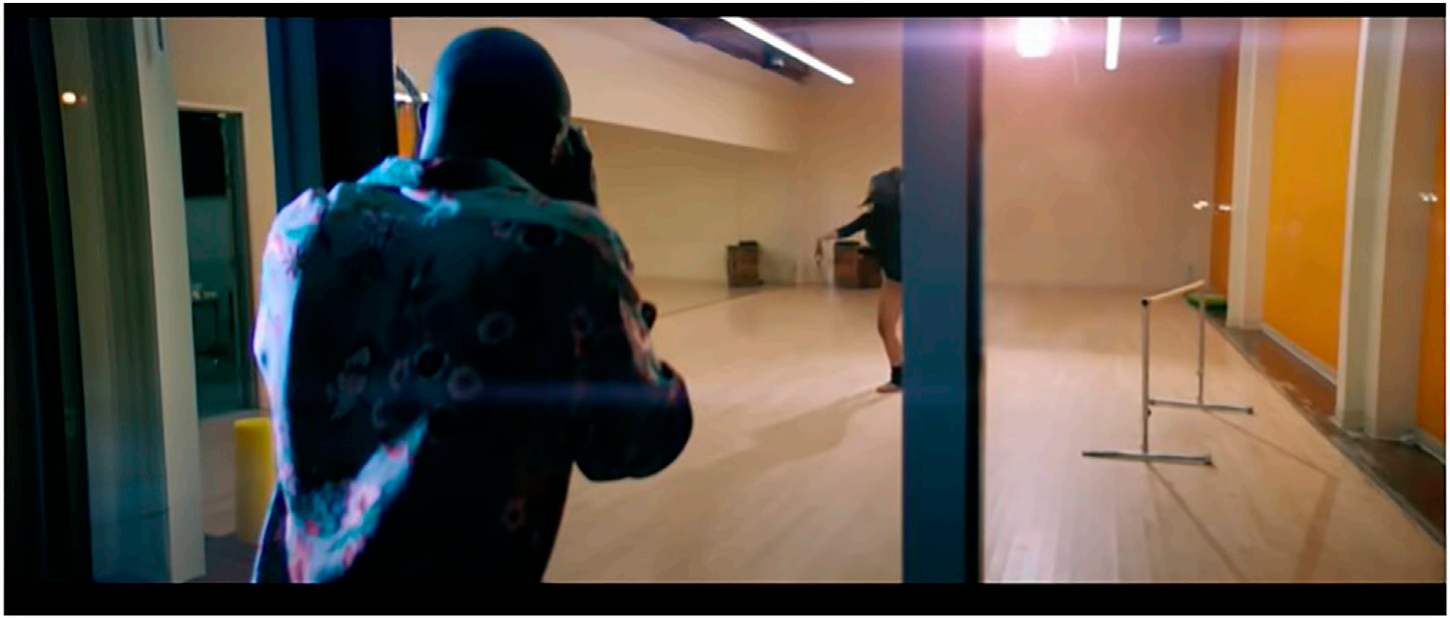
Afrobeats artist, Davido, depicted as voyeuristically watching a woman dancing in a well-lit dance studio [screenshot taken from *Fall (Official Music Video):* https://www.youtube.com/watch?v=3Iyuym-Gci0].

Consider Figure 3, from the music video for the song “Fall” by Davido (2017b). The female character featured in the video–wearing only a black bodysuit and ankle socks–is subjected to a voyeuristic (male) gaze that actively situates Davido (the gazing subject) at a distance which allows him to enjoy a pleasure in seeing. That pleasure is derived, firstly, from being able to see and potentially fetishize what is being seen. Secondly, pleasure is derived from the fact that the person being gazed upon is unaware of this gaze and may not threaten the gazer by returning the gaze. In the video, Davido is physically distancing himself from the woman and in sheer voyeuristic fashion, he is ‘hidden’ in the darkness of the evening while the woman being looked at is illuminated by the light in the dance studio she is inside of.

Through these visio-spatial organisations of these Afrobeats music videos as short filmic narratives, particular relational ties between men (masculinities) and women (femininities) are constructed and sustained. One significant relational ideology sustained here, is that women’s right to privacy is less valued, and thus exposes them to situations of being more prone to violation in their unjust position as ancillary sexual objects. Having grappled with these visual portrayals, it is apparent that the representational conventions in the visual culture captured by the analyzed Afrobeats music videos, sustain partly misogynistic gender relations that thrive on the hetero-erotic objectification of women’s bodies specifically for a voyeuristic black African male gaze. These gender relations are implicated in particularly disempowering effects to women. It is disempowering to women in that, the depicted women’s bodies are significantly positioned as lacking in terms of being multifaceted, culturally influential and able to embody leadership as opposed to servitude. This, as burgeoning feminist scholarships have theorized, disempowers women and strips them off their agency as it relates to their expressions of sexuality and femininity.

Considered through a post-feminist lens, however, notions of women’s dis-empowerment as a result of powerful (heterosexual) desiring male gazes, are notably disrupted in how the diverse African women featured in the selection of analyzed music videos appear to actively use their sexualities and femininities in various scenes to exercise considerable power over the protagonist men co-featured in these music videos. These featured women engage in what may be read as an active claiming of their sociocultural power over restrictive patriarchal conditions, especially in the domains of (heterosexual) intimacy and romantic gender relations. As such, considerably pervasive in the corpus of analyzed music videos, are young, attractive, heterosexual African woman who are depicted as “knowingly and deliberately play [ing] with [their] sexual power” (Gill, 2008: 41) in ways that see them partly exercise control over the featured (domineering) men in the diverse hyperreal narratives characterized by the plot progressions in these music videos. These expressions of feminine sexuality and femininity are closely aligned with what Gill (2008) describes as a postfeminist-aligned ‘midriff’ sensibility, as discussed in the sub-section below.

### Post-Feminist Media Cultures and the “Midriff” Sensibility

Theorized as knowingly using her body, appearance, and sexuality to exercise her (sexual) agency more assertively, the post-feminist-entangled “midriff” woman is described as unapologetic about her sexual power and ability to (freely) use her body as a means to socioculturally, and otherwise, advance or benefit herself. So notes Gill (2008: 41):

The midriffs might be thought of as a generation of girls and young women in their teens and 20's in the 1990's, but midriff also refers to a *sensibility* characterized by a specific constellation of attitudes toward the body, sexual expression and gender relations.

Tracing a midriff sensibility in advertising media, Gill (2008) notes four central themes characterizing this cultural sensibility. These four themes, Gill suggests, are “an emphasis upon the body, a shift from objectification to sexual subjectification, a pronounced discourse of choice and autonomy, and an emphasis upon empowerment” (2008: 41). With respect to its emphasis on the body, the midriff sensibility places considerable value on women’s bodies as their key sociocultural capital. In contemporary media cultures, “it is now possession of a “sexy body” that is presented as women’s key source of identity” (Gill, 2008: 42). Her success and attractiveness are deemed viable when she convincingly self-manages her body to maintain it in a suitably toned, waxed, scented and dressed (or perhaps under-dressed) condition (Bordo, 1993; Gill, 2008).

Secondly, the ways in which women’s bodies are represented in (often semi-erotic texts) in popular media forms, had also experienced a notable shift. “Where once sexualized representations of women in the media presented them as passive, mute objects of an assumed male gaze, today women are presented as active, desiring sexual subjects who choose to present themselves in a seemingly objectified manner because it suits their (implicitly “liberated”) interests to do so” (Gill, 2008: 42). Crucial to these two cultural shifts as they relate to women and their bodies, is an explicit linkage thereof to notions of freedom and choice on the part of women. Herein, women in popular media forms are presented as knowingly negating men’s approval and instead doing things to please and benefit themselves, “and, in so doing, they “just happen to win men’s admiration”” (Gill, 2007: 42; Gill, 2008).

I briefly focus on the plot progression in one of the analyzed music videos to demonstrate traces of a midriff-aligned feminine expression by the protagonist woman character. The central plot around which the narrative in the music video for the song “Collabo” by P-Square (2015) and Don Jazzy develops, is that of an infatuation-driven pursual–by three men (portraying the three protagonists)—of the same woman who appears to be their superior at their depicted workplace. This woman is portrayed in a multifaceted manner where she is depicted as a powerful professional, managing the company for which these men work. She is, for instance, depicted in boardroom scenes that indicate a sense of influence and superiority embodied through how she “leads” the meetings in said boardroom scenes. Even so, she is still sexually objectified through several camera shots that frame an explicit focus on her buttocks, thighs and cleavage area as the music video progresses.

The three protagonist men engage in several attempts at catching this woman’s attention to their advances without any form of intimidating coercion. As such, she may be read as not directly undermined or stripped of her agency to actively reject these men’s advances–communicating, implicitly, a sense of non-hostility from the men who are evidently behaving in ways that may be deemed emasculating depending on which lens of hegemonic masculinity you view it through. The woman remains in control of her sexuality and status of power, and eventually uses the very sexual advances made by all three men to lure them into doing some of her work-based tasks that seem to have piled up, given her presumed senior position in the portrayed company. She is depicted, then, as quite powerful and self-determining in her reliance on her physical and sexual attractiveness–regarded in post-feminist, midriff-aligned feminine performances as social capital (Gill, 2008)—to benefit her in her professional progression.

Additionally, also central to a midriff cultural sensibility–alongside notions of agentic choice and pleasing of the self on the part of women–is the discursive thematic of female/feminine empowerment. Gill (2008) demonstrates how a discourse of feminine empowerment prevails in how advertising targeted at women creatively “sell” the promise of self-esteem and confidence. Women are interpellated in contemporary advertising, aligned along a midriff sensibility, as powerful in their sexualized femininities. Power in their sexualized femininities means that women who internalize a midriff sensibility are encouraged to recognize their sexual power “to bring men to their knees” (Gill, 2008: 43). There is a particular “power femininity” that thrives within post-feminist cultural formations that reassures women that “feminist struggles have ended,” and complete equality for all women is an achieved reality; therefore, women can “have it all”, should they so choose (Lazar, 2006; Gill, 2007; Gill, 2008).

Quite similar to the storyline depicted in the “Collabo” music video analyzed earlier, the plot line for the “Chop My Money” music video by P-Square (2012), also partly revolves around several men’s pursuits of the same woman. The artists featured on the song–Paul, Peter, Akon and May-D–portray the protagonist men in romantic pursuit of the same woman, although without their knowledge. In pursuit of this woman, the four men are portrayed as engaging in quite explicit forms of romanticism often mediated by various forms of “breadwinnerism” and wealth expending. I borrow the term “breadwinnerism” from sociological studies that have used it to capture the normative expectation on men–in a dominant patriarchal cultural formation–to be financial and material providers (Akanle et al., 2014; Alliyu and Adedeji 2016). The central female character–spotting a toned figure, flawless skin and cleavage close-ups in several scenes–is, as a result, the recipient of unrestricted luxurious material commodities (luxury cars, expensive shopping sprees and access to premium credit cards) from each of these men in their attempts at securing her romantic interest.

In a very self-determining fashion, this woman is depicted as agentic in her eventual decision to outright reject all four of the men pursuing her, without any hostile consequences that may expose her to any form of harm from any of the men involved. The men respectfully accept their fates because, upon finding out that this woman “played” all of them by not giving into any of their advances while enjoying all the materialistic “perks” and eventually choosing to be with a different man altogether, they collectively acknowledge their misfortune, and thus leave the woman be. At the end of this music video, many a viewer would agree that the protagonist woman in this music video manged to successfully “…bring [those] men to their knees” (Gill, 2008: 43). Her agency and control over her body, thus, are not threatened, one may argue. Thereby positioning her, in the minds of the viewer, as empowered and confident enough to use her sexual power as a means to ‘have it all’ (Gill, 2008).

Further “empowered femininity” can also be read in the narrative progression for the music video, ‘No kissing’, by Patoranking (2016) and Sarkodie. This music video’s plot revolves around a young heterosexual couple in love. The protagonist man, played by Patoranking, and his romantic partner are quite evidently in what seems to be a loving relationship. Patoranking even goes as far as claiming to be fully appreciative of this love to such an extent that he behaves quite “gentleman-like” in that he exercises extreme self-restraint with regard to his sexual urges toward his love interest. He communicates this by assuring the woman that for as long as she refuses to engage in sexual activity with him, he will not force her into it. Consider this excerpt of lyrics from the song:

If you no gimme [give me], I no go take

Only your love I appreciate

If you no gimme, I no go take

Baby girl let's relate

Without actually saying it outright, Patoranking, with these lyrics, assures the woman that if she is not going to “give” him, he is not going to take. That is to say, he will practice self-control instead of forcefully ‘taking’ with regards to sexual intercourse. If it is only her love she has to offer now, that is fine with him–he appreciates her love. While implicitly quite misogynistic in its underlying insinuation that, as a man, he *should* be “given” what he wants from a woman and should thus be celebrated for not forcefully taking it from her; it is the superficial sentiment veiling the misogyny that may be read as romantic in that he may be seen as going against the grain of violent masculinities that would have typically *demanded* intimacy from the woman. It would seem, in this case, that the woman is rendered a very accommodating space to agentically determine the rules/expectations in this relationship because she confidently dictates that, in this relationship, there shall be:

No kissing baby

No touching baby

No kissing baby

And don't call me baby

Here, it is apparent, that the woman is depicted as being in control of what happens to her body in the domains of sexuality and intimate relations. Thereby, arming her with “power femininity” (Gill, 2008: 43).

Having demonstrated how gender relations are depicted in the multimodal discourses of a select few Afrobeats music videos, I will now show how the dialectic gender-relational sensibility of misogyrom–that I propose underscores these gendered depictions in the analyzed music videos–offers an empowering potential as it relates to women through these hyperreal depictions. However, I wish to argue, these readings of women’s empowerment, agency and self-determination, are more of a social façade effectively mediated and pseudo-legitimized by discourses of extreme romanticism in tandem with a post-feminist sensibility which–if taken at face value–may encourage the uncritical absolving of heteropatriarchy from its deeply toxic nature toward women’s actual sociocultural advancement and just treatment in the domains of sexuality, intimacy and romantic relationships. Essentially, the *actual* power remains firmly in the hands of men.

## The Façade of the Empowered, Agentic and Self-Determining Woman in Afrobeats Music Videos: The Intersection of Post-Feminism and Misogyrom

Misogyrom as a gender-relational sensibility pervasive in the corpus of Afrobeats music videos analyzed in this research paper, variously mediates the constructions of femininities in these texts; as it does masculinities per my arguments earlier in the paper. As an underlying sensibility across the diverse gender relations depicted in the analyzed music videos, a misogyrom sensibility is effective in fostering flexible ground for the expression of what may appear to be post-feminist aligned, self-determining femininities. Due to its cultivation of romantic and compassionate gender-relational atmospheres (while still inherently misogynistic in its cultural superiorizing of men), misogyrom allows room for the pushback against the rigidity of patriarchy in its largely denigratory treatment of women compared to men, even in the context of intimate heterosexual relationships and expression of sexuality.

Sustained through active, and perhaps inadvertent, performances of ideal “manhood” that conceal misogyny and sexism with overtly courteous acts of romanticism that, on the surface, appear to be void of violence and hostility, a misogyrom sensibility appears to render women a “safe space” to assert their agency, across the domains of intimacy and sexual expression. The femininities featured in the analyzed texts assert their perceived agency by, for instance, being depicted as confidently assessing, rejecting and generally being critical of the courtship attempts from the “parading” masculinities who, as chauvinistic patriarchal norms would dictate, would typically resort to exercising dominance by forcefully convincing women into accepting their courtship advances.

Here, interestingly, despite the arrogant visual and lyrics-based emphasis on their wealth, physical “sexiness” and strength, as well as their self-proclaimed sexual prowess, the diverse masculinities pervasive in the corpus of music videos analyzed, are constructed as particularly *not* demanding control over the attention and affection of the women to which they are pleading romantic love. They come across, especially through their lyrical content, as not expressing entitlement over women’s bodies. This is an observation that may be appreciated by several pockets of feminist scholars (Sommers-Flanagan et al., 1993; McRobbie, 2004; Bretthauer et al., 2007; Mowatt et al., 2013; Ligaga, 2014; Ligaga, 2020 etc) who have partly spoken truth to power as it relates to heteropatriarchal men and their relationships with women’s bodies.

Being variously implicated in misogyrom, the masculinities centralized in the analyzed music videos, are saliently depicted as exerting overt emotional labor in pursuit of romantic relations with the women they wish to court. This emotional labor is partly also manifesting as “peacocking” behavior that these men engage in. Peacocking, as some relationship “experts” explain through circular and popular discourse, is something men do so as to emphasize what they perceive are their strong attributes in order to stand out from their ‘competition’ in their pursuits to attract women (Hayes, 2019). In so doing, the peacocking man invests significant emotional labor while trying to convince his love interest as to why he is the best choice of romantic partner.

It is in this egotistical parading of themselves as “ideal” lovers and “providers,” that these masculinities portrayed in the analyzed music videos, appear to engage in symbolic forms of self-auctioning which may come across as though the critical and sexualized gaze and its inherent power is shifted from masculine subjectivities to feminine subjectivities. This is so in that the (powerful?) feminine subjectivities, in such instances of men’s peacocking, are invited to temporarily bear the active gaze as targets of the self-auctionineering done by the men. In the context of the visual data, the female characters actively pursued and serenaded by the peacocking male characters in the analyzed music videos, may appear as if temporarily empowered in that they are seemingly rendered an examinatory gaze to counter-objectify these men. The counter-objectification could be perceived to exist in moments when the courted woman actively “assesses” her peacocking pursuer to make a decision on whether or not he is worthy of her attention and affection. Once having “decided” on a suitable suitor, the women featured in many of the music videos analyzed, are overtly assured of their value as partners through romantic discourses of infinite love, affection and fidelity, communicated by the protagonist men in these videos through their song lyrics, especially. The women are showered with romanticism and are made to feel they can, for instance, confidently dictate that there shall be “no kissing” and “no touching” (or sexual activity) until such a time when *they* are ready. This relates to post-feminist sensibilities which appear to afford women the freedom to be assertive in their sexualities. Just by this sheer sense of agency afforded them in regard their bodies, they may be seen as somewhat empowered in the domains of sexuality and intimate relations involving their bodies.

What, then, does one make of this complex dialectic of misogyrom intersecting with post-feminist sensibilities, at play in the visual culture of the Afrobeats music videos analyzed, where women are quite evidently treated with respect, adoration and an atmosphere for sexual agency on the one hand; while equally denigrated to disempowering positions of oversexualized object of men’s erotic pleasure? A useful pursuit, I’m convinced, is to seek out where the power *actually* lies in these misogyrom sociocultural relations–variously implicated in post-feminist sensibilities–among men and women as depicted in the analyzed selection of music videos. That way, a more reflexive observation can be made about potentially how far the multiplicity of feminist agendas toward equal power division among men and women in the domains of sexuality and intimate relationships, among others, have come, and how effective have feminist discourses been in inspiring the young, cosmopolitan youths participating in the production of these popular cultural texts in contemporary Africa, to promote gender-relational equality and the just treatment of women in the domains of sexuality, intimacy and romantic relationships. In that vein, I argue that, regardless of the evident alignment with post-feminist formations about empowered women, the power still firmly lies in the hands of the men featured in these music videos in their capacities as cultural chaperones of heteropatriarchy.

This is most evident in two ways. Firstly, it is the featured men who remain in positions of dominance over women and their sexual expressions by non-coercively encouraging the sexualization of women in how they sing about these women’s “assets” (buttocks), for instance. For Davido (2014) to, for example, “romantically” instruct his love interest to gyrate her buttocks for his pleasure, and she obliging to the request; he wields power over her. This is a level of power that may go unnoticed because of how effectively Davido veils it with overly romanticized expressions of love, fidelity and adoration toward the woman.

Secondly, we have seen that the analyzed masculinities thrive on asserting their dominance and superiority by overtly parading their wealth, social status and positions as ideal breadwinners. It is in these narcissistic expressions of masculinities that these featured men co-construct femininities that are shown as voluntarily taking on gender roles of (sexual) servitude and willing beneficiaries of men’s provision, rescue out of economic constraint, and sexual satisfaction. The sub-text of such gender-relational depictions then, once again, places sociocultural power squarely in the hands of men.

To help sum up my argument, I briefly draw on a study undertaken by Dunu and Ugbo (2015) in Nigeria, focusing on the music lyrics of Afrobeats artist, Flavor, as they relate to women and their bodies. In a focus group discussion about the nature of Flavor’s musical lyrics in regard to women, a female respondent alluded to an interesting observation which captures particularly what my theorization of misogyrom partly proposes. As Dunu and Ugbo (2015: 46) aptly summarise:

The women pointed out that although most of Flavor’s songs tend to adore, praise and make women prominent; it only creates false pretence of empowerment that only serves to secure the woman for the man’s satisfaction and fulfilment.

It is such observations that help us see the toxic nature of romanticism in its attempt to guize misogyny and thereby re-assert heteropatriarchal, sexist social cues to debase women through a “false pretence of empowerment” in the domains of sexuality and intimate relationships.

## Conclusion

This article has placed focus on Afrobeats; a globally popularizing musical genre of African origin. This musical genre is widely consumed by transnational audiences; resulting in the music videos produced by artists from this scene, reaching YouTube viewership figures ranging in the hundreds of millions. As such, this article considered these Global South popular cultural texts as valuable conduits through which to critically examine contemporary social constructions and performances of gender and politically charged meanings of “womanhood” in relation to “manhood” as implicated as they are in heteropatriarchal collective African cultural worldviews. The paper situated this musical genre’s multimodal discourses, specifically as they relate to gender and sexuality, within a broader post-feminist discourse about women’s (sexual) empowerment, agency and self-determination.

By first offering a critical reading of performances of masculinities in the corpus of nine music videos, the article was able to demonstrate the catalytic nature of masculine performativities on performances of “womanhood”/femininities in these music video texts. Masculine performativities, I have argued, are implicated in a misogyrom sensibility which fosters gender-relational expressions that are at once implicated in the perpetuation of misogynistic imbalanced gender power relations and undeniable hyper-romanticism and compassion from (heterosexual) men toward women. As such, expressions of “manhood” in the analyzed music videos are sustained along inherently sexist and misogynistic atmospheres of men’s superiority and dominance while (in)advertently encouraging the egalitarian treatment of women by lyrically valuing them as more than mere objects of men’s sexual satisfaction, but romantic partners with value more than that which a patriarchal male gaze had historically often relegated to *just* physical appearance. What this does, also, is allow a post-feminist sensibility to appear valuable and actively at-work in how the women are partly depicted.

In that vein, I argue that, regardless of the evident alignment with post-feminist formations about empowered women, the sociocultural power still firmly lies in the hands of the men featured in these music videos in their capacities as cultural chaperones of heteropatriarchy. It is the featured men who–through consistent misogyrom–remain in positions of dominance over women and their sexual expressions by non-coercively encouraging the hypersexualization of women in how these men sing about these women’s bodies. Additionally, the featured men assert their dominance and superiority by overtly parading their wealth, social status and positions as ideal breadwinners in the analyzed music videos. It is in these narcissistic expressions of masculinities that these featured men co-construct femininities that are shown as voluntarily taking on gender roles of (sexual) servitude and willing beneficiaries of men’s provision, rescue out of economic constraint, and sexual satisfaction. The sub-text of such gender-relational depictions then, once again, places sociocultural power squarely in the hands of men. By veiling these sexist advances with hyper-romanticism, the featured men implicitly debase women through a “false pretence of empowerment” (Dunu and Ugbo 2015) in the domains of sexuality and intimate relationships, as mediated in the corpus of music videos analyzed in this study.

I argue, then, that traces of post-feminist cultural sensibilities in tandem with a misogyrom sensibility–encouraging a reading of women as empowered and agentic in the analyzed music videos–are regressive for the feminist advancement of gender-relational equality. This is so because these sensibilities implicitly encourage misogyny to prevail through the iteration of regressive patterns of behavior that uphold (hetero)patriarchy in these popular cultural texts. Therefore, readings of empowered black African women–as positive progress in the plight toward equal gender power relations in a notably heteropatriarchal African popular cultural imaginary–should be celebrated with caution, as they may derail the feminist plight toward women’s *actual* (sexual) empowerment across their diverse African contexts in and beyond popular media texts.

## Data Availability

The original contributions presented in the study are included in the article/Supplementary Material, further inquiries can be directed to the corresponding author.
